# Modality-specific quantitative radiologic characterization of diffuse sclerosing osteomyelitis of the mandible: a pilot study

**DOI:** 10.1007/s00784-026-07160-8

**Published:** 2026-10-02

**Authors:** Katharina Theresa Obermeier, Wenko Smolka, Sven Otto, Anusha Abdullah, Gabriele Kaeppler, Eric Hesse, Ina Dewenter, Yoana Malenova

**Affiliations:** 1 Department of Oral and Maxillofacial Surgery and Facial Plastic Surgery, LMU University Hospital, LMU Medizin, Ludwig-Maximilians-Universität München, Lindwurmstr. 2a, D-80337 Munich, Germany; 2 Institute of Musculoskeletal Medicine, University Hospital, LMU Munich, Fraunhoferstraße 20, 82152 Planegg-Martinsried, Germany; 3 Musculoskeletal University Center Munich, University Hospital, LMU Munich, Fraunhoferstraße 20, 82152 Planegg-Martinsried, Germany

**Keywords:** Diffuse sclerosing osteomyelitis, Imaging, Cone beam CT, Bone density, Panoramic radiography

## Abstract

**Objectives:**

Diffuse sclerosing osteomyelitis (DSO) of the mandible is a rare chronic inflammatory disease characterized by recurrent painful swelling and trismus. While advanced stages are known to show radiographically evident bone sclerosis, objective quantitative imaging criteria remain poorly defined. This study aimed to characterize modality-specific radiological features of DSO using intra-individual side-to-side comparison of bone density and cortical thickness.

**Materials and methods:**

This single-center retrospective study included 22 patients treated for DSO between 2010 and 2021. Pre-treatment panoramic radiographs were available for all patients, CT datasets for 6 patients, and CBCT for 3 patients. Bone density was quantified using relative grayscale values in panoramic radiography, Hounsfield units (HU) in CT, and gray values in CBCT. Cortical thickness was measured on CT using standardized regions of interest, with contralateral-side comparison. Two blinded observers performed all measurements.

**Results:**

Quantitative analysis demonstrated significantly higher bone density on the affected side in panoramic radiography and CT (panoramic: 46.0 ± 4.0% vs. 39.0 ± 3.6%, *p* < 0.001; CT: 1220.9 ± 215.8 HU vs. 1108.0 ± 163.9 HU, *p* = 0.01). In the three available CBCT datasets, descriptively higher gray values were observed on the affected side. CT analysis revealed significantly increased cortical thickness (5.63 ± 0.96 mm vs. 3.86 ± 0.75 mm, *p* = 0.04). A blurred inferior alveolar nerve canal was consistently observed on panoramic radiographs.

**Conclusions:**

Quantitative side-to-side assessment provides an objective radiological description of DSO and may support imaging assessment when interpreted together with clinical and histopathological findings.

**Clinical relevance:**

In this selected cohort, DSO-affected regions showed relative densification, cortical thickening in the available CT examinations, and blurring of the inferior alveolar nerve canal on panoramic radiographs.

## Introduction

Diffuse sclerosing osteomyelitis (DSO) is a poorly understood, presumably chronic inflammatory disease of the jaw. The exact trigger and pathogenesis of the disease remain unclear. Some authors advocate the hypothesis that DSO is initiated, at least in part, by an infection that leads to a chronic inflammatory process [1]. It is considered a subtype of primary chronic osteomyelitis (PCO). DSO is found in the jawbone, with a predilection for the mandible. The disease mostly affects adults, with a higher incidence in women [2].

Patients present with symptoms of local swelling, hyperthermia, and pain [3]. Patient-reported pain ranges from 8 to 10 on the visual analog scale [4]. The disease occurs in an episodic fashion, with symptomatic episodes often followed by asymptomatic intervals [5]. Radiographically, DSO commonly presents as a mixed sclerotic and osteolytic lesion with a solid periosteal reaction or external bone resorption [3]. In later stages, areas of bone sclerosis become more frequent.

Treatment of DSO is discussed controversially in the literature. In former times, when the behavior and nature of DSO were less clearly understood, surgical treatment was performed and is still performed according to Jia et al. (2023) [11]. However, drug therapy approaches such as steroids, analgesics, antibiotics, and hyperbaric oxygen therapy also exist [1, 6]. Besides these treatment options, therapy with antiresorptive drugs has also been described in the literature [7, 8]. Otto et al. (2015) were the first to describe a case series of 10 patients treated with ibandronic acid who experienced significant pain reduction immediately after treatment. For instance, pamidronic acid and ibandronic acid have been successfully used to treat acute inflammatory episodes of DSO [4]. In 2018, Otto et al. reported the treatment of a patient with DSO using denosumab. They considered it an alternative treatment for patients suffering from DSO. This led to the assumption that antiresorptive drugs may not only stabilize and positively modulate bone metabolism but also exert anti-inflammatory effects [9].

However, the most challenging part is establishing the diagnosis of DSO in daily practice, since various chronic bone lesions can resemble one another clinically in terms of symptoms and disease onset. Histopathological diagnosis is difficult and, with regard to differentiation from chronic suppurative osteomyelitis (CSO) and craniofacial fibrous dysplasia, often inconclusive [10, 11]. Because the pathophysiology of other chronic bone conditions differs from that of DSO, an accurate diagnosis is of utmost importance.

Imaging plays a central role in the diagnostic workup. Panoramic radiography and three-dimensional imaging modalities, such as computed tomography (CT) and cone-beam computed tomography (CBCT), are routinely used in clinical practice. While their general diagnostic value is well established, objective and reproducible imaging-based characteristics that may support the diagnosis of DSO have not been systematically defined [12, 13]. In particular, quantitative assessment strategies must account for the distinct physical measurement principles of each imaging modality.

The aim of the present study was therefore to identify modality-specific radiological characteristics of DSO using intra-individual side-to-side comparison within each imaging modality. By focusing on relative differences between the affected and contralateral, clinically and radiologically unaffected mandibular regions, this approach aims to provide reproducible imaging parameters while avoiding direct cross-modality quantitative comparisons. Such characterization may facilitate a more objective radiological assessment of DSO when interpreted together with clinical and histopathological findings. Because DSO was not directly compared with other sclerosing conditions in the present study, these parameters describe DSO rather than establishing their ability to discriminate it from such entities.

## Materials and methods

This single-center retrospective study was approved by the institutional review board of the University Hospital of Munich, Germany (Munich, Germany; UE Nr. 22–0445). The study includes 22 patients who were treated at our hospital between 2010 and 2021. Only patients with a diagnosis of diffuse sclerosing osteomyelitis and available pre-treatment panoramic radiography were included. Because no universally accepted diagnostic gold standard exists for DSO, diagnosis was established through multidisciplinary evaluation integrating clinical presentation, radiological findings, and histopathological assessment where available. Typical clinical features included recurrent mandibular pain, swelling, and episodic disease activity. Radiological findings were evaluated for characteristic features of DSO, including diffuse sclerosis, cortical thickening, mandibular enlargement, and periosteal bone formation.

Alternative diagnoses, including chronic suppurative osteomyelitis, fibrous dysplasia, ossifying fibroma, and other fibro-osseous lesions, were excluded based on clinical history, imaging findings, and histopathological examination. Histopathological specimens were available in 16 of 22 (72.7%) patients and were reviewed alongside the clinical and radiological findings. In the remaining six patients without histopathological confirmation, the diagnosis of DSO was established by clinical and radiological follow-up.

Where available, CBCT and CT datasets were additionally analyzed.

Demographic data, lesion localization, lesion size, date of primary diagnosis, clinical symptoms, type of therapy (surgical or non-surgical using anti-resorptive medication), and average follow-up time were recorded. Patients with other bone diseases, including primary and secondary osteoporosis, or with other forms of osteomyelitis, were excluded. All imaging data analyzed in this study were obtained before initiation of therapy.

### Panoramic radiography

All panoramic radiographs were acquired using Orthophos XG (Sirona Dental System). Bone density measurements were performed using Sidexis 4 (Dentsply Sirona, Charlotte, North Carolina, USA) and expressed as relative percentage values according to the software’s internal grayscale scaling. To further validate the findings, measurements were repeated using Fiji (ImageJ, National Institutes of Health, Bethesda, MD, USA), and mean gray values (GV) were recorded. Density measurements were performed only as intra-individual comparisons between the affected and contralateral mandibular side within the same radiograph.

Within the radiographically defined DSO region, ten measurement points were evenly distributed across the affected bone area. These points were placed within the trabecular bone of the mandibular body, below the apices of the teeth and above the inferior cortical border, and spread along the extent of the lesion to sample its full area rather than a single focus. In the four patients whose DSO was localized to the temporomandibular joint region rather than the mandibular body, the measurement points were placed within the affected region instead of the corpus, again sampled against the corresponding contralateral region. For intra-individual comparison, ten mirrored reference points were placed on the contralateral, clinically unaffected mandibular side. If mirrored points overlapped with anatomical structures of differing density (e.g., tooth roots or cortical borders), they were slightly adjusted to remain within trabecular bone. Because panoramic magnification and geometric distortion affect both hemimandibles comparably, grayscale values were interpreted only for intra-individual comparisons between the affected and contralateral sides within the same radiograph, rather than as absolute values. Lesion length and width were recorded. The inferior alveolar nerve canal was assessed qualitatively for visibility and delineation.

All measurements were performed independently by two observers blinded to each other’s results. Interobserver reliability was assessed using Pearson correlation. Panoramic density measurements represent relative grayscale information and were interpreted only in comparison to the contralateral side within the same radiograph.

### Computed tomography

High-resolution CT scans of the facial skeleton (slice thickness 0.625 mm) were analyzed. DICOM datasets were imported into Mimics (Materialise, Leuven, Belgium). Mineralized bone tissue was segmented using the predefined Bone (CT) threshold preset, with a lower threshold of 226 Hounsfield units (HU). No upper threshold was applied, so that hyperdense, highly sclerotic bone was fully retained within the available Hounsfield range.

####  Definition of the region of interest (ROI)

After segmentation, the mandible was separated into right and left hemimandibles along the median plane. The ROI comprised the entire mandibular hemibody corresponding to the diseased side and the contralateral side; thus, two ROIs were defined per patient (affected vs. contralateral hemimandible). Teeth and the mandibular canal were excluded from segmentation to avoid artificial elevation of density values. Cortical and cancellous bone were not analyzed separately but evaluated as a composite mineralized structure within the defined ROI. Mean HU values were calculated from all voxels within each ROI. Boundary voxels potentially affected by partial volume effects were minimized by threshold-based segmentation and exclusion of non-mineralized structures. No normalization or transformation of CT-derived HU values was performed.

####  Cortical thickness measurement

Cortical thickness was measured at three standardized vestibular points per hemimandible: the anterior border of the lesion, the posterior border of the lesion, and the midpoint between these two locations. The three measurements obtained per hemimandible were first averaged to yield a single mean cortical thickness value per side for each patient, and these per-patient side means were then compared between the affected and contralateral reference sides. All CT density measurements were interpreted strictly within the CT modality and as intra-individual side-to-side comparisons.

###  Cone beam computed tomography (CBCT)

Available CBCT datasets were analyzed descriptively. Gray values were extracted from the segmented mandibular hemibodies using the same anatomical ROI definition as applied in CT analysis. It is acknowledged that CBCT gray values are device-dependent and do not represent standardized or calibrated HU. Therefore, CBCT-derived values were used exclusively for intra-individual side-to-side comparison and were not directly compared with CT-derived HU values. No cross-modality normalization was performed because the physical measurement principles and output parameters differ fundamentally across imaging techniques.

### Statistical analysis

Statistical analysis was conducted using SPSS (version 24, IBM Corp., Armonk, NY, USA). Data distribution was assessed using the Shapiro-Wilk test. Normally distributed data are presented as mean ± standard deviation (SD), non-normally distributed data are presented as median and interquartile range. As the affected and contralateral sides represent paired measurements within the same individual, paired t-tests were used to compare quantitative parameters. A p-value < 0.05 was considered statistically significant.

Given the limited availability of CBCT datasets, results were analyzed descriptively rather than with inferential statistics due to the small sample size.

## Results

### Demographic data, clinical symptoms, and treatment

DSO of the mandible is a rare condition with limited cohort data available. Overall, 22 patients were included in this study. Sixteen patients (72.7%) were female and six (27.3%) were male. The mean age at diagnosis was 43.6 ± 20.0 years (range 23–63 years). The localization of DSO was in the mandibular corpus in 18 patients and in the temporomandibular joint in 4 patients.

All patients reported recurrent episodes of severe mandibular pain accompanied by swelling. No trigger factor or initiating event could be identified in any of the patients. For all patients, medical records documented a long-standing disease with various prior treatments that provided only temporary symptom relief.

All patients had used non-steroidal anti-inflammatory drugs during severe pain episodes. Twenty-one patients received a single intravenous dose of ibandronic acid (6 mg) during an active phase of the disease, and one patient received a single subcutaneous dose of denosumab (60 mg). Laboratory parameters were assessed before antiresorptive therapy.

Evaluation of general medical conditions revealed arterial hypertension in one patient, who was treated with ramipril 1.25 mg once daily. None of the other patients received long-term medication.

Pre-treatment panoramic radiographs were available for all 22 patients, CT datasets for 6 patients, and CBCT datasets for 3 patients.

### Panoramic radiography

**Fig. 1:**
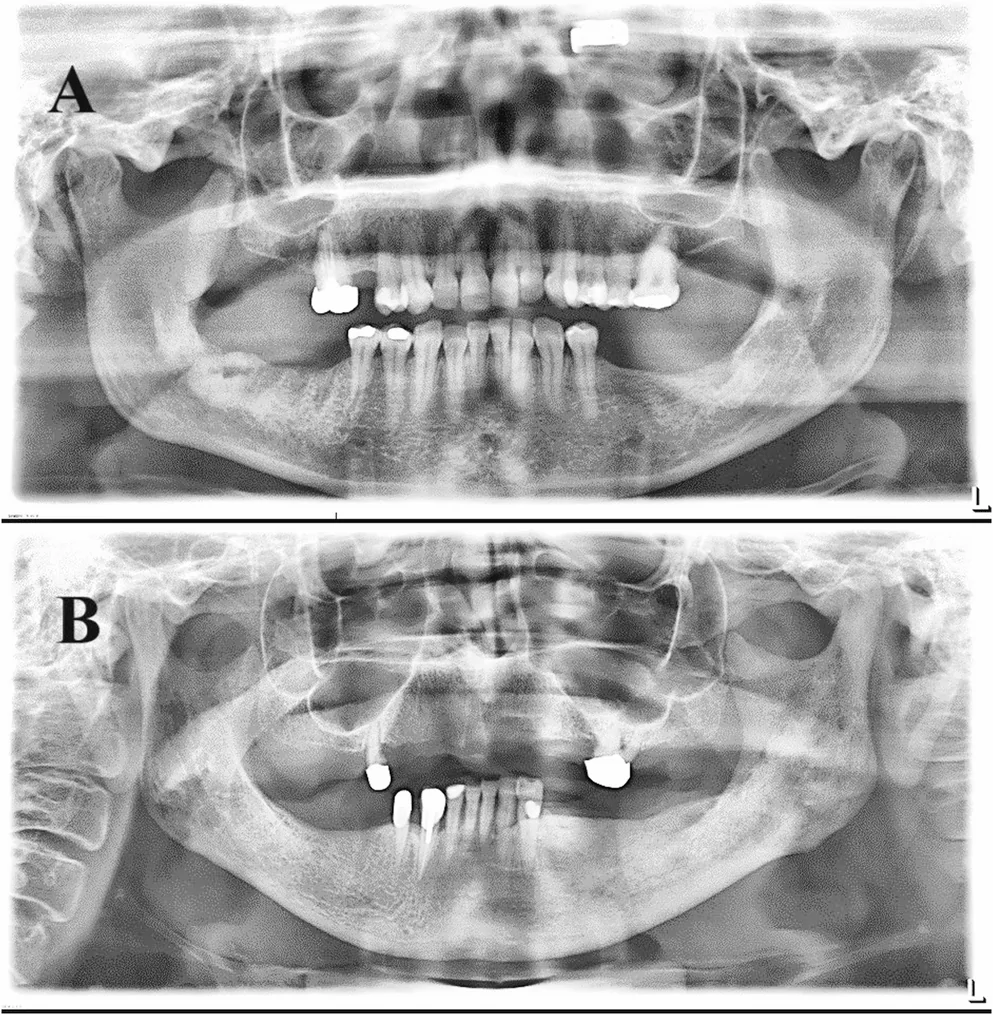
Panoramic radiographs showing blurred inferior alveolar nerve canals in patients with DSO (*n* = 22). (**A**) blurred nerve canal on the right side, (**B**) blurred nerve canal on the left side

Panoramic radiography remains a first-line modality for assessing mandibular structural changes in DSO, particularly for evaluating cortical integrity and neurovascular canal delineation. Pre-treatment panoramic radiographs were available for all 22 patients. Pathological fractures or sequestra were not detected in any case. Condylar process deformation was observed in 4 patients (18.2%). In all cases, the inferior alveolar nerve canal appeared blurred or poorly delineated on the affected side (Fig. 1).

#### Density measurements

Quantitative assessment of mandibular bone density in DSO remains limited, particularly regarding intra-individual side-to-side differences. Using Sidexis software, mean relative density values on the affected side were 46% ± 4.01%, compared to 39 ± 3.61% on the contralateral side. ImageJ analysis yielded mean gray values of 120.21 GV on the affected side and 98.91 GV on the contralateral reference side. In paired statistical analysis, density values were significantly higher on the affected side compared to the contralateral non-affected side for both measurement methods (paired t-test, *p* < 0.001).

Interobserver reliability ranged from moderate to high. Pearson correlation coefficients were 0.61 (affected side) and 0.46 (contralateral reference side) using Sidexis, and 0.96 (affected side) and 0.75 (contralateral reference side) using ImageJ.

Panoramic density measurements represent relative grayscale differences within the same radiograph and were interpreted only in comparison to the contralateral side. Individual bone density values are shown in Table 1.

**Table 1:** Relative density values (%) of DSO-affected vs. contralateral reference side in panoramic radiography (*n* = 22)

Patient number	Average density values DSO-affected side	Average density values contralateral reference side
1	41%	35%
2	43%	37%
3	44%	36%
4	48%	37%
5	43%	37%
6	43%	41%
7	45%	38%
8	44%	40%
9	44%	39%
10	44%	34%
11	54%	44%
12	51%	45%
13	46%	37%
14	53%	44%
15	40%	34%
16	43%	37%
17	50%	40%
18	44%	40%
19	45%	40%
20	50%	45%
21	52%	38%
22	43%	32%

### CBCT

**Fig. 2:**
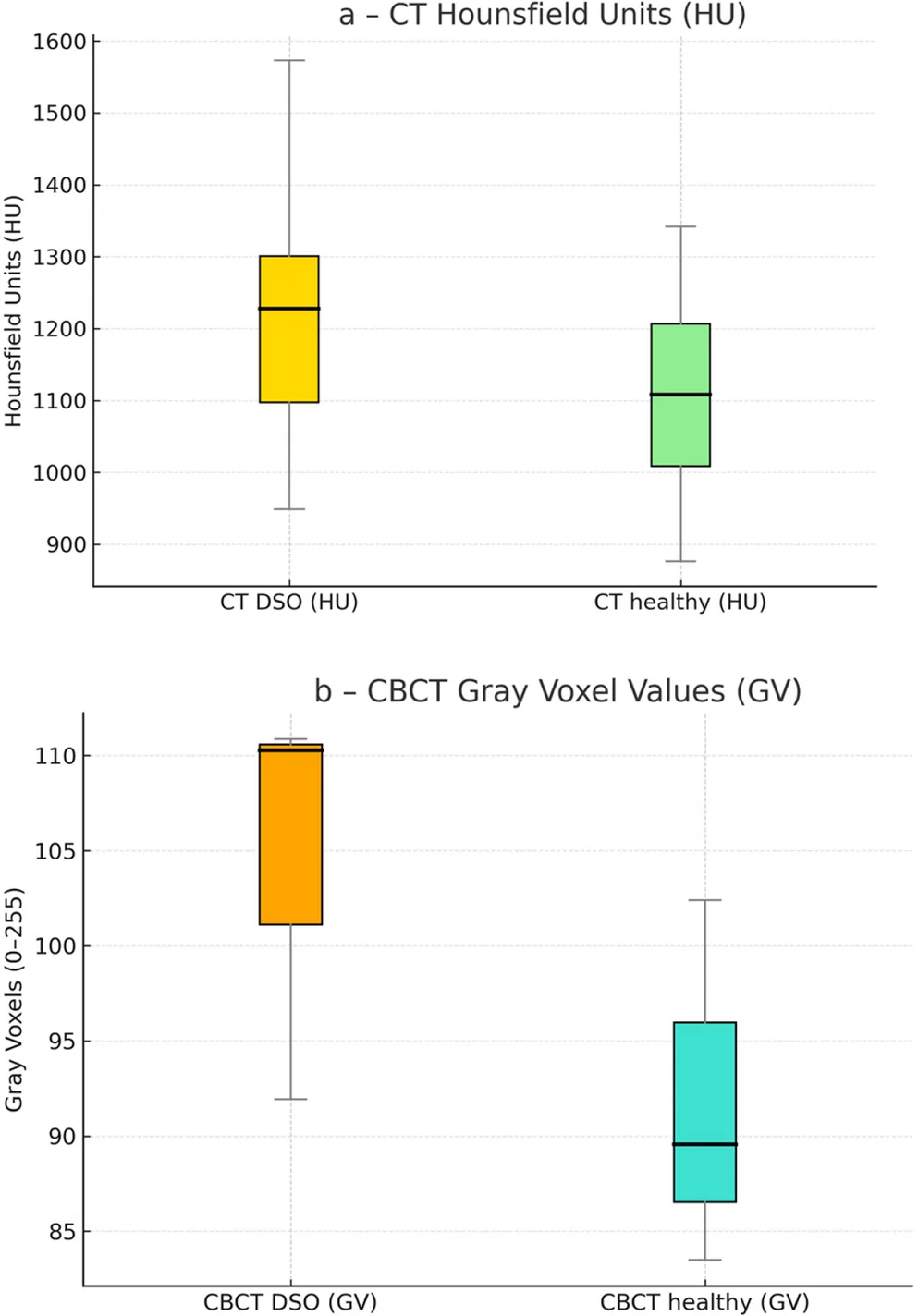
(**a**) Boxplot showing the distribution of average Hounsfield units in DSO-affected and contralateral reference sides as measured by CT. (**b**) Boxplot of gray voxel values in DSO-affected and contralateral reference sides derived from CBCT. CT *n* = 6, CBCT *n* = 3

CBCT was used as an additional imaging modality to explore side-to-side differences in mandibular bone density. CBCT datasets were available for three patients. Gray values were analyzed descriptively using the predefined anatomical regions of interest. In all three cases, higher gray values were observed on the DSO-affected side compared to the contralateral side. Given the limited sample size and device-dependent nature of CBCT gray values, no inferential statistical analysis was performed. CBCT results are presented descriptively in Table 2; Fig. 2b. These observations should be interpreted cautiously given the very limited sample size.

**Table 2:** CBCT-derived bone density values (gray voxel values) between the DSO-affected and contralateral reference sides (*n* = 3)

DSO side (GV)	Healthy side (GV)
110.867	102.396
110.276	83.488
91.934	89.560

### CT

CT datasets were available for six patients and were used for quantitative assessment of intra-individual side-to-side differences in bone density and cortical morphology.

#### Density analysis

**Fig. 3:**
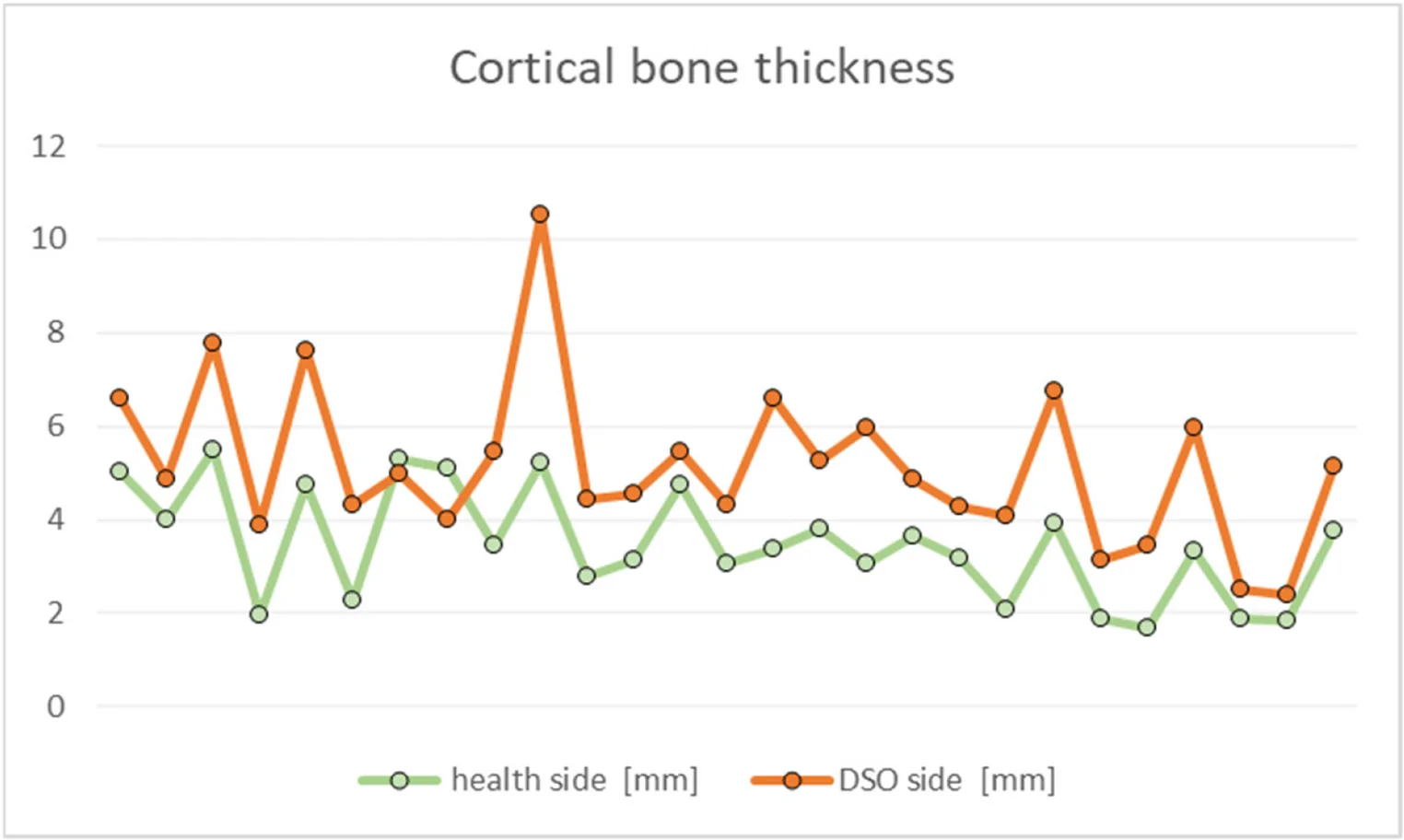
Comparison of cortical bone thickness between DSO-affected and contralateral reference sides based on CT measurements (*n* = 6)

Mean HU were higher on the affected side (1220.85 HU ± 215.75 HU) than on the contralateral reference side (1108.03 HU ± 163.85 HU). Paired statistical analysis demonstrated a significant intra-individual difference between affected and contralateral healthy sides (paired t-test, *p* = 0.01). Results are summarized in Table 3 and illustrated in Figs. 2a and 3.

**Table 3:** CT-derived bone density values (Hounsfield units) between the DSO-affected and contralateral reference side (*n* = 6)

DSO side Bone density (Average HU)	Healthy side Bone density (Average HU)
1238.202	1155.517
948.8696	876.284
1227.699	1073.767
1363.203	1257.204
1573.207	1341.817
974.803	943.6314

#### Cortical thickness

Mean cortical bone thickness measured 5.63 ± 0.96 mm on the affected side and 3.86 ± 0.75 mm on the contralateral reference side. Paired analysis revealed a statistically significant increase in cortical thickness in DSO-affected regions (*p* = 0.04).

## Discussion

DSO is still a poorly understood chronic inflammatory disease of the mandible. It is considered a form of chronic osteomyelitis, and patients typically present with painful mandibular swelling, often accompanied by trismus and a history of prior tooth extraction [14]. Mostly women are affected, and the average age has been reported to be relatively young, which is comparable to our cohort, although DSO can occur in all age groups [14]. As also documented in our patients, DSO is difficult to diagnose, and individuals are often referred from doctor to doctor for years before a definitive diagnosis is made. In particular, the differentiation between chronic suppurative osteomyelitis, DSO, fibrous dysplasia, ossifying fibroma, and bacterial osteomyelitis is often challenging, as these entities can present with similar clinical complaints. Because DSO remains a diagnosis of exclusion, it is inherently difficult to establish. Radiological, clinical, and histopathological findings indicate that DSO closely resembles chronic suppurative osteomyelitis and craniofacial fibrous dysplasia [14]. Because the radiographic appearance of DSO overlaps with that of other chronic mandibular bone diseases, clinical parameters — including patient age, the presence and episodic, recurrent pattern of pain, functional impairment such as trismus, and the duration of symptom onset — remain essential discriminators and should be weighed together with the imaging pattern described here when differentiating DSO from entities such as chronic suppurative osteomyelitis, fibrous dysplasia, and ossifying fibroma. Moreover, the histopathological distinction between DSO and fibrous dysplasia can be difficult, particularly if the GNAS mutation, which is characteristic of fibrous dysplasia, cannot be confirmed [15]. Although DSO has traditionally been considered a diagnosis of exclusion, diagnostic confidence in our cohort was strengthened by integrating clinical, radiological, and histopathological findings. Histopathological specimens were available in 16 of 22 patients.

Given these diagnostic challenges, imaging plays a central role in the diagnostic process. However, objective and reproducible radiological criteria for DSO have not been systematically defined. The present study, therefore, aimed to identify modality-specific imaging characteristics by performing intra-individual side-to-side comparisons between the affected and contralateral mandibular sides. Given the limited sample size, this study should be interpreted as an exploratory pilot study aimed at generating quantitative imaging hypotheses rather than providing definitive diagnostic criteria.

CT allows quantitative assessment of radiodensity using HU, which reflects tissue attenuation relative to calibrated reference materials [1]. Air is defined as −1000 HU and water as 0 HU, while bone tissue typically ranges from 1000 to 3000 HU [16]. A correlation between HU and bone mineral density has been reported, and normal jawbone has been classified into density groups to estimate its physical properties by imaging. Bone with the highest density has been described as having HU values above approximately 1250 [16]. In addition, previous work has demonstrated that trabecular bone volume strongly correlates with bone density and mechanical resistance [17]. In the present study, CT analysis demonstrated significantly higher HU values on the DSO-affected side compared to the contralateral side. This finding is consistent with the clinically and histopathologically described sclerosis in DSO. Similarly, panoramic radiographs showed significantly higher relative density values on the affected side when compared intra-individually with the contralateral reference side. In our cohort, these findings indicate that DSO was associated with increased relative bone density compared with the contralateral mandible on each modality examined, provided that interpretation remains strictly modality-specific. However, these findings should be interpreted with caution due to the limited number of available CT and CBCT datasets.

It is important to emphasize that CT-derived HU and CBCT gray values represent fundamentally different physical parameters [18]. CBCT gray values are device-dependent and not standardized. Therefore, density analyses were interpreted strictly within each imaging modality and limited to intra-individual side-to-side comparison. No cross-modality quantitative comparison was performed. For this reason, the three available CBCT cases were evaluated descriptively and not included in the quantitative three-dimensional density analysis.

Panoramic radiography, although it does not provide calibrated density values, demonstrated consistent relative grayscale differences between the affected and contralateral sides. These findings suggest that even two-dimensional imaging can reveal relative densification in DSO when evaluated comparatively within the same radiograph. Taken together, increased bone density relative to the contralateral side appears to be a recurring radiological feature of DSO on the modalities examined – robustly on panoramic radiography and CT, and descriptively in the three available CBCT cases - when interpreted within their respective modalities.

Typical radiological characteristics of patients with DSO include extensive cortical and medullary sclerosis, subperiosteal bone formation, and deformation of the condylar process [19]. In addition to densification, CT analysis demonstrated significantly increased cortical thickness on the affected side. Cortical thickening likely reflects chronic inflammatory remodeling and subperiosteal bone formation, both of which are characteristic features described in DSO. These findings support the concept of hyperostotic remodeling as a structural hallmark of the disease. Our findings demonstrated significantly increased radiographic density in the affected mandibular regions compared with the contralateral side, consistent with the sclerotic nature of DSO and prior radiological descriptions in the literature. The increased radiographic density observed in affected mandibular regions should not be interpreted as a disease-specific finding, as sclerosis may also occur in several other inflammatory and fibro-osseous conditions. Rather, the value of the present analysis lies in the quantitative assessment of radiological characteristics that have previously been described predominantly in qualitative terms. Therefore, increased density should be considered in conjunction with other imaging features, including cortical thickening, mandibular enlargement, periosteal bone formation, and blurring of the inferior alveolar nerve canal. The combination of these findings provides a more objective description of the radiological appearance of DSO. Whether this pattern improves discrimination from other chronic mandibular bone diseases requires direct comparative studies.

CT scans also illustrated variability in the extent of hyperostosis and sclerosis, with the most pronounced patterns observed in DSO-affected regions. Involvement of the cortical bone results in thickening and, in some cases, a loss of the cortical-medullary border, rendering the cortical bone indistinguishable from cancellous bone [19]. In our study, cortical thickness was significantly higher on the affected side than on the contralateral side (5.63 ± 0.96 mm vs. 3.86 ± 0.75 mm), further corroborating a hyperostotic pattern.

A blurred or poorly delineated inferior alveolar nerve canal was consistently observed on the affected side in panoramic radiographs and may reflect inflammatory remodeling of the surrounding cancellous bone. While not pathognomonic, this finding appears to be a recurring qualitative feature in DSO. Enlargement and morphological alteration of the inferior alveolar nerve canal in DSO have also been reported previously [10, 20]. To definitively distinguish between different disease entities, histopathological samples, microbiological findings, and imaging results must be evaluated in combination [10, 11].

To improve the radiological characterization of DSO, we aimed to describe quantitative and qualitative imaging features across routinely used imaging modalities. CT and CBCT are considered more sensitive to changes in bone mineralization than panoramic radiography [13, 21]. However, typical imaging features on panoramic radiographs, including sclerosis, cortical irregularity, mottling, radiolucent components, and a blurred nerve canal, can indicate DSO, although these features are characteristic of DSO rather than specific to it. Although panoramic radiography provides less information on absolute bone density than CT, it involves substantially lower radiation exposure. Despite its limitations in density measurement, our results showed comparable trends, with densification of the affected bone in the sense of sclerosis. Overall, we identified bone density measurements and cortical thickness as quantifiable markers, whereas sclerosis and blurring of the inferior alveolar nerve canal represent qualitative characteristics. The combination of relative densification, cortical thickening, and blurring of the inferior alveolar nerve represents a recurring radiological pattern observed in our cohort. From a practical standpoint, when DSO is considered in the differential diagnosis, panoramic radiography can serve as an accessible first-line tool: relative densification and cortical thickening together with a blurred inferior alveolar nerve canal on the affected side, assessed against the contralateral side, may raise suspicion for DSO and should be interpreted together with the clinical presentation and, where indicated, histopathological findings. Where available, CT or CBCT may then show cortical thickening, always interpreted as an intra-individual side-to-side comparison and in conjunction with the clinical course and histopathology. Importantly, density measurements must be interpreted as relative intra-individual findings rather than absolute values, particularly when different imaging modalities are used. Imaging findings alone are insufficient to establish the diagnosis. Still, they may substantially contribute to diagnostic confidence when interpreted in conjunction with clinical presentation and histopathological assessment.

This study has several limitations. First, the retrospective design and limited sample size restrict the generalizability of our findings. However, DSO is a rare condition, and comparable case series in the literature report similarly small cohorts [7, 22]. Although panoramic radiographs were available for all 22 patients, CT datasets were available for only 6 patients, and CBCT datasets for only 3 patients. Therefore, CT-derived HU measurements, cortical thickness assessment, and CBCT-based gray value comparisons should be interpreted with caution. The present study should be regarded as exploratory and hypothesis-generating rather than confirmatory. A further limitation relates to panoramic radiography. As a projection-based imaging modality, panoramic imaging is subject to geometric distortion, magnification effects, and superimposition of anatomical structures. These factors may influence grayscale measurements. To reduce such effects, density assessment was performed exclusively as an intra-individual side-to-side comparison within the same radiograph rather than as an absolute comparison between different patients. Several factors, including heterogeneous trabecular bone patterns, gradual transitions between affected and unaffected bone, superimposition effects inherent to panoramic radiography, and variability in ROI placement, may explain the moderate interobserver agreement observed in this study. Finally, magnetic resonance imaging (MRI) was not systematically available in our cohort. MRI might provide additional information about bone marrow edema and soft-tissue involvement, particularly in early disease stages. Future prospective studies with larger cohorts and standardized multimodal imaging protocols are warranted to further refine radiological criteria for DSO. Furthermore, the present data do not permit the definition of absolute diagnostic thresholds; deriving modality-specific normative cut-off values would require larger, prospectively standardized cohorts.

Because imaging contributed both to the diagnostic work-up - including the exclusion of alternative diagnoses – and to the radiological outcomes analysed here, a degree of incorporation bias cannot be excluded; the quantitative parameters should therefore be regarded as a description of a cohort already diagnosed with DSO rather than as independent diagnostic criteria. All included patients had unilateral disease, and the contralateral side was considered clinically and radiologically unaffected and used as the intra-individual reference.

In the selected cohort, higher relative values were statistically confirmed on panoramic radiography and CT, while higher grayscale values were descriptively observed in the three available CBCT scans. These findings provide a quantitative description of the imaging appearance of DSO in this cohort. The ability to distinguish DSO from other mandibular diseases remains unknown.

## Conclusions

DSO remains a diagnostic challenge due to overlapping clinical and radiological features with other chronic mandibular bone diseases. In this retrospective exploratory study, DSO-affected regions showed three recurring imaging findings, each evaluated on the modality suited to it and interpreted only as an intra-individual side-to-side comparison:

1. blurred delineation of the inferior alveolar nerve canal on panoramic radiographs (*n*=22),
2. increased bone density relative to the contralateral reference side on panoramic radiography (*n*=22) and CT (*n*=6).
3. cortical thickening of the affected hemimandible on CT (*n*=6).

Quantitative density assessment should be interpreted strictly within each imaging modality and as an intra-individual comparison. Cross-modality quantitative comparison is not appropriate due to fundamental differences in physical measurement principles. Recognition of these imaging features may facilitate the radiological assessment of patients with suspected DSO when interpreted together with clinical findings and histopathological evaluation. Future comparative studies are required to determine whether these features can reliably distinguish DSO from other chronic mandibular bone diseases.

## Data Availability

The data that support the findings of this study are not publicly available due to confidentiality reasons. The anonymized statistical data is available from the corresponding author (KTO) upon reasonable request.
